# Supplementary material for: Protocol for an implementation study of an evidence-based home cardiac rehabilitation programme for people with heart failure and their caregivers in Scotland (SCOT:REACH-HF)
Source: BMJ Open. 2020 Dec 4;10(12):e040771. doi: 10.1136/bmjopen-2020-040771 (PMC7722379; doi:10.1136/bmjopen-2020-040771)
Supplement: Supplementary data [file bmjopen-2020-040771supp001.pdf]

APPENDIX 1 - SCOT:REACH-HF Interview Topic Guide

Introduction:

- Explain interview purpose / study aims
- Emphasise no right/wrong answers, can decline to answer at any time
- Opportunity for questions
- Check consent, permission to record

General:

- Tell me a little about yourself (e.g. Job title, key roles/responsibilities)

Starting out with REACH-HF

- What was your first impression of REACH-HF?
- Tell me about your experience of the facilitator training (if applicable)
- Was the training sufficient to enable you to deliver REACH-HF? (*PROMPT anything you would have liked to see done differently, added/left out, additional resources?)*
- Was there anything that made it easier/more difficult for you to take part in the training?
- To what extent do you feel you started with a clear understanding of what you were being asked to do?

Implementing REACH-HF (facilitators)

- Tell me about when you first began delivering the intervention *(PROMPT first patient session e.g. initial experiences, concerns, information gaps, confidence)*
- How has delivering REACH-HF differed from your usual way of working?
- Was there anything that made it easier/more difficult for you to deliver the intervention? *(PROMPT adequate support)*
- What did you see as being the main purpose of REACH-HF? *(PROMPT has that changed?)*
- What, if any, changes did you make to how you delivered the intervention as time went on (e.g. to suit your way of working, or the patient's needs)? *(PROMPT for details)*
- How did you find the task of completing the post-session checklist (after recorded sessions)? *(PROMPT e.g. useful, additional burden etc)*

Implementation of REACH-HF (non-facilitators)

- *Tell me about your impressions of how the delivery of REACH-HF went in your area*
- *Was there anything that made it easier/more difficult for REACH-HF to be delivered in your area?*
- *Can you tell me about any additional resources that were needed for REACH-HF? Or changes in roles/responsibilities?*

Embedding REACH-HF

- How, if at all, has delivering REACH-HF changed the way you work? *(PROMPT changes specific to home self-care; changes to way team works; if expect likely to be lasting change)*

- How easy has it been to integrate REACH-HF into your usual work? *(PROMPT good fit or not)*
- What changes, if any, would you make to REACH-HF to suit your way of working?
- Of those you work with, who is it that's driven REACH-HF forward? *(PROMPT role, what they've done to support delivery; can include self)*
- In what way, if any, has implementing REACH-HF changed working relationships in your team?
- Is there anything else that would make it easier for REACH-HF to become part of routine practice for your team? *(PROMPT additional skills, training, support)*
- Are your team evaluating the impact of REACH-HF on your service? *(PROMPT details)*
- What do you think is likely to be the future of REACH-HF in your service? *(PROMPT likely to become routine practice?)*

Overall impressions of REACH-HF

- To what extent do you feel that offering the intervention has been worthwhile?
- When you've discussed as a team how REACH is going, can you tell me about how those conversations have gone? *(PROMPT similarities/differences around e.g. aims, expected benefits as a mode of delivery)*
- What, if anything, do you see as being the value of the intervention:
    - For you
    - To your patients (if applicable)
- Has the COVID-19 pandemic impacted your delivery of REACH-HF/CR?
- What are your overall views on REACH-HF? (PROMPT anything not already noted)
- Is there anything else you think it is important for the research team to know?

Close

- Any questions?
- Feedback on interview?
- Thank participant and close.
