# Supplementary material for: Protocol for an implementation study of an evidence-based home cardiac rehabilitation programme for people with heart failure and their caregivers in Scotland (SCOT:REACH-HF)
Source: BMJ Open. 2020 Dec 4;10(12):e040771. doi: 10.1136/bmjopen-2020-040771 (PMC7722379; doi:10.1136/bmjopen-2020-040771)
Supplement: Supplementary data [file bmjopen-2020-040771supp002.pdf]

APPENDIX 2 – PROJECT MANAGEMENT GROUP AND INDEPENDENT ADVISORY GROUP MEMBERSHIP

Project Management Group *(To oversee the progress and delivery of the project)*

Prof Rod Taylor - Chief Investigator, University of Glasgow

Dr Carrie Purcell – Project Manager, University of Glasgow

Dr Hayes Dalal – Co-Applicant, REACH-HF co-Chief Investigator / Honorary Clinical Associate Professor, University of Exeter / Senior Clinical Researcher, Royal Cornwall Hospitals NHS Trust

Dr Clare Murphy - Co-Applicant, NHS Greater Glasgow & Clyde / Scottish National Advisory Committee for Heart Disease – Heart Failure Subgroup Chair and Clinical Lead

Dr Aynsley Cowie – Co-Applicant, Consultant Physiotherapist in Cardiology, NHS Ayrshire & Arran / BACPR Council Member

Dr Tracy Ibbotson – Co-Applicant, Patient and Public Involvement and Engagement Lead, College of Medical, Veterinary and Life Sciences, University of Glasgow

Prof John Cleland – Co-Applicant, Robertson Centre for Biostatistics, University of Glasgow

Mrs Claire Kerr – Robertson Centre for Biostatistics, University of Glasgow

Project Advisory Group *(To provide independent advice and direction to the project)*

Ms Frances Divers – Cardiac Rehabilitation Champion, NHS Scotland (chair)

Dr Edwin Jesudason – Cardiac Rehabilitation Clinical Lead, NHS Scotland

Mr Richard Forsyth – Health Services Engagement Lead, British Heart Foundation Scotland

Mr Nick Hartshorne-Evans – CEO, Pumping Marvellous (patient group)

Ms Louise Taylor – Head of Services, Heart Manual Department, NHS Lothian

Dr Hayes Dalal – REACH-HF co-Chief Investigator / Honorary Clinical Associate Professor, University of Exeter / Senior Clinical Researcher, Royal Cornwall Hospitals NHS Trust

Ms Helen Wilson, Head of Research, Heart Research UK (observer)
